# Integrated serum metabolomics and network pharmacology reveal molecular mechanism of Qixue Huazheng formula on peritoneal fibrosis

**DOI:** 10.3389/fphar.2025.1515038

**Published:** 2025-01-23

**Authors:** Xiaohui Meng, Li Sheng, Yongqing You, Huibo Dai, Manshu Yu, Funing Wang, Ziren Zhou, Yun Shan, Meixiao Sheng

**Affiliations:** ^1^ Department of Nephrology, Affiliated Hospital of Nanjing University of Chinese Medicine, Nanjing, China; ^2^ Medical Research Center of First College of Clinical Medicine, Nanjing University of Chinese Medicine, Nanjing, China; ^3^ Department of Nephrology, Kunshan Affiliated Hospital of Nanjing University of Chinese Medicine, Kunshan, China

**Keywords:** traditional Chinese medicine, Qixue Huazheng formula, peritoneal fibrosis, metabolomics, network pharmacology, estrogen signaling, ras signaling, apoptosis

## Abstract

**Background:**

Peritoneal fibrosis (PF) causes peritoneal dialysis (PD) withdrawal due to ultrafiltration failure. Qixue Huazheng formula (QXHZF), comprising *Astragalus membranaceus*, *Centella asiatica*, and *Ligusticum sinense*, is applied to treat PD-related peritoneum injury related; however, the active components, core genes, and underlying mechanism involved remain unclear.

**Methods:**

The anti-PF effects of QXHZF were verified *in vivo* and *in vitro*. Targets underlying QXHZF-mediated improvement of PD-induced PF were predicted using network pharmacology analysis. Metabolites associated with QXHZF treatment of PD-related PF were analyzed by serum metabolomics. Integration of network pharmacology and serum metabolomics findings identified potentially important pathways, metabolites, and targets, and molecular docking studies confirmed the interactions of key components and targets. Western blotting (WB), quantitative real-time PCR (qRT-PCR), TdT-mediated dUTP Nick-End Labeling (TUNEL) staining, and flow cytometry were conducted.

**Results:**

QXHZF had potent therapeutic efficacy against PF according to WB, qRT-PCR, and pathological section examination. Network pharmacological analysis indicated that multiple QXHZF compounds contributed to improving PF by modulating various targets and pathways. Differential metabolites were identified by serum metabolomics analysis. Integrated data analysis indicated that steroid hormone biosynthesis, the Ras signaling pathway, apoptosis, and estrogen signaling contributed to the effects of QXHZF. Metabolite-target network and molecular docking analyses revealed that QXHZF can bind to estrogen receptor 1 (ESR1) and rapidly accelerated fibrosarcoma 1 (RAF1) through its components. WB demonstrated that QXHZF treatment reversed activation of the above-mentioned signaling pathways, thereby inhibiting PD fluid-induced PF.

**Conclusion:**

QXHZF can significantly ameliorate PD-induced PF and may regulate estrogen signaling, the Ras pathway, and apoptosis in this context.

## 1 Introduction

Peritoneal dialysis (PD) is recommended as the preferred renal-replacement therapy regimen for end-stage renal disease (ESRD) because it provides good protection of residual renal function; approximately 11% of patients with ESRD undergo PD (Teitelbaum, 2021). Nevertheless, withdrawal of long-term PD is often associated with ultra-filtration failure, related to peritoneal anatomic alterations, such as fibrosis. One mechanism underlying peritoneal fibrosis (PF) involves chronic exposure to biologically incompatible PD fluids (PDF), which contain high concentrations of glucose and glucose degradation products (Bajo et al., 2017). The peritoneum (PM) comprises a single layer of mesothelial cells on submesothelial interstitial tissue, and is consequently susceptible to mesothelial-to-mesenchymal transition (MMT) attributable to PDF. In MMT, mesothelial cells abandon epithelial characteristics and develop mesenchymal features that contribute to PF (Masola et al., 2022). To date, no effective methods to treat PF are available; therefore, we sought effective treatments for this condition.

Due to its characteristic features of having multiple components that influence numerous targets, Chinese herbal medicine has excellent therapeutic potential and scientific research value (Li et al., 2021). In Traditional Chinese Medicine (TCM) theory, the core pathogenesis of PF depends on spleen and kidney deficiency, dampness turbidity, and blood stasis. With increased PD duration, the PM is exposed to PDF for long periods, which weakens healthy qi and leads to retention of local qi and blood, as well as stasis in the abdominal collaterals. According to the theory of “Kidney Qi”, developed by Yanqin Zou, a master of TCM, clinical treatments for PF generally include qi invigoration, detoxification, and blood activation. *Astragalus membranaceus* (Chinese name: Huangqi) can tonify qi, generate blood, promote blood circulation, and clear stasis (Gong et al., 2022). The effects of *Centella asiatica* (Chinese name: Jixuecao) include detoxifying, activating blood, clearing heat, and draining dampness (Biswas et al., 2021). In addition, *Ligusticum sinense* (Chinese name: Chuanxiong) promotes qi and blood, as well as alleviating pain and expelling wind (Lin et al., 2022). Thus, Qixue Huazheng formula (QXHZF), which consists of Huangqi, Jixuecao, and Chuanxiong, based on the theoretical foundations of TCM and our previous research (Dai et al., 2023), possessing numerous pharmacological impacts, including antioxidant, anti-inflammatory, and anti-fibrosis effects, as well as regulation of angiogenesis (Yu et al., 2018; Zhao et al., 2020; Zhu et al., 2021). How QXHZF improves PF is currently unclear; thus, further research is crucial to comprehensively and deeply dissect the mechanism underlying its effects.

Network pharmacology is an effective tool to connect the complex components involved in TCM with molecular level mechanisms (Zhang et al., 2020). Network models can be constructed to reveal relationships among ingredients, targets, and diseases, and are commonly applied to predict the potential targets of Chinese herbal medicine components. The recent emergence of metabolomics has made it possible to determine the metabolic mechanisms involved in biological systems (Pan et al., 2020). Therefore, the combination of network pharmacology and serum metabolomics is a potentially effective approach to elaborate the mechanisms underlying the effects of QXHZF on PF.

**FIGURE 1:**
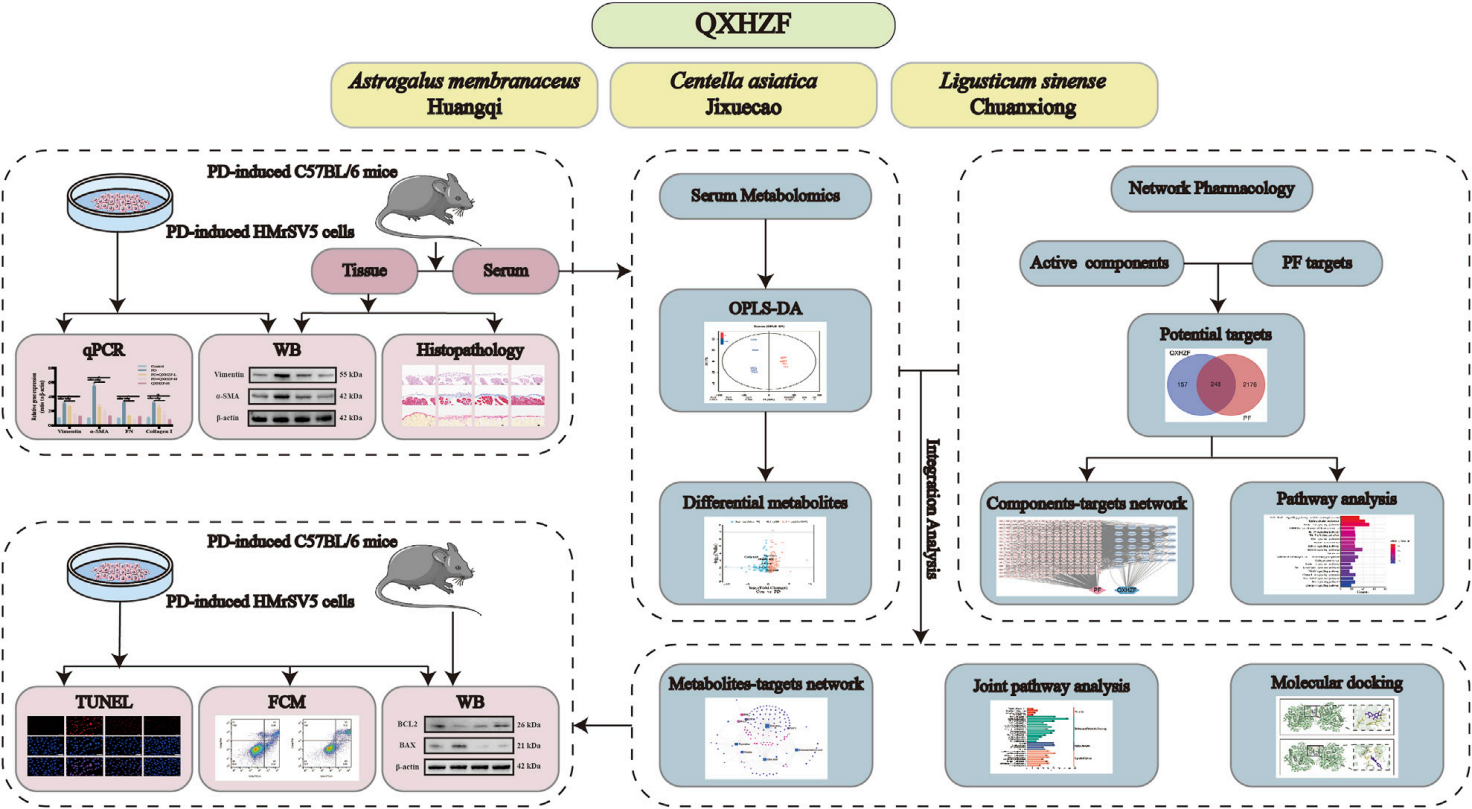
Workflow for investigating the mechanisms underlying the effects of QXZHF treatment on PD-induced PF.

In this study, the pharmaco-activity of QXHZF in treating PF was first verified, both *in vivo* and *in vitro*. Subsequently, network pharmacology analysis was conducted to identify the bioactive components in QXHZF, in terms of targets and pathways. In addition, serum metabolomics analysis was performed, to investigate the changes in endogenous metabolites induced by PDF, with or without QXHZF treatment. Further, we integrated the data generated by network pharmacology and serum metabolomics to filter important signaling pathways. Finally, key targets in these pathways were experimentally assessed, to provide convincing evidence of the potential mechanisms underlying QXHZF treatment and TCM theory (Figure 1). This is the first study to clarify the therapeutic mechanisms underlying the effect of QXHZF on PF using a combined network pharmacology and serum metabolomics approach.

## 2 Materials and methods

### 2.1 Experimental validation

#### 2.1.1 Preparation of QXHZF and QXHZF-containing serum

QXHZF, consists of *A. membranaceus* (Chinese name: Huangqi) (30 g), *C. asiatica* (Chinese name: Jixuecao) (15 g), and *L. sinense* (Chinese name: Chuanxiong) (15 g) granules (Jiangyin Tianjiang Pharmaceutical Co., Ltd., Jiangyin, China), which were purchased from the Affiliated Hospital of Nanjing University of Chinese Medicine. For animal experiments, granules were dissolved according to the above ratio in double-distilled water (ddH2O). QXHZF-containing serum was prepared for use in cell experiments. SD rats (6–8 weeks, 200–250 g, male) were purchased from Vital River Laboratory Animal Technology Co., Ltd. (Beijing, China). Rats were treated with 6.3 g/kg QXHZF by oral gavage once daily for 7 consecutive days. The dosage was calculated according to the coefficients of the equivalent dose for humans and animals by body surface area in <Pharmacological Experimental Methodology>, as follows:

$$\text{clinical dose for humans}=60\;g\;\left(30\;g\text{ Huangqi},15\;g\text{ Jixuecao},\times 15\;g\text{ Chuanxiong}\right)/60\text{ kg}$$

dose for SD rats=60 g/ 60 kg×70 kg×0.018/0.2 kg=6.3 g/kg

Rats in the Control and model groups received an equal amount of saline daily. One hour after the end of administration, rats were anesthetized for collection of blood from the abdominal aorta in negative pressure procoagulant tubes. Samples were centrifuged at 3,000 × g for 15 min after clotting at room temperature for 1 h. The serum was then heat-inactivated at 56°C for 30 min in a water bath, filtered through a 0.22 μm filter to removal bacteria, and stored for use in cell experiments.

#### 2.1.2 Animal experiments

C57BL/6J mice (6–8 weeks, 20–25 g, male) were purchased from Vital River Laboratory Animal Technology Co., Ltd. (Beijing, China). Mice were adaptably fed for 7 days and randomly divided into four groups (n = 6 mice per group): Control, PD, PD + QXHZF-L, and PD + QXHZF-H. Mice in the PD, PD + QXHZF-L and PD + QXHZF-H groups were intraperitoneally injected with 0.1 mL/10 g PDF (Baxter, Guangzhou, China) in 4.25% dextrose daily for 28 days to establish the PF model. The equivalent dose for C57BL/6J mice was calculated as follows:

60 g/60 kg×70 kg×0.0026/0.02 kg=9.1 g/kg

Mice in the PD + QXHZF-L and PD + QXHZF-H groups were treated daily with 4.55 g/kg/d and 9.1 g/kg/d QXHZF respectively by oral gavage for 28 days (for the same duration as PD modeling), and mice in Control and PD groups received an equal amount of saline daily. Each mouse was weighed twice a week. At the end of the experiment, mice were anesthetized for collection of blood and peritoneal tissue.

#### 2.1.3 Cell culture and intervention

Human peritoneal mesothelial cells (PMCs; HMrSV5) were purchased from Shanghai Lian Mai Bioengineering Co., Ltd. (Shanghai, China) and cultured in Roswell Park Memorial Institute 1,640 (RPMI-1640) medium (Thermo, MA, United States) supplemented with 10% (v/v) fetal bovine serum (HomelandBio, Nanjing, China) and 1% (v/v) penicillin/streptomycin (Thermo, MA, United States) in a 5% CO_2_ humidified incubator at 37°C. HMrSV5 cells were seeded, grown for 24 h to reach 75% confluence, and then pre-treated with medium supplemented with 5%, 10% QXHZF-containing or control serum. Then, cells were incubated with 3% PDF to induce the PF model for a further 24 h.

#### 2.1.4 Cell viability

The cell counting kit-8 (CCK8) assay was applied to assess the toxicity of QXHZF-containing serum in HMrSV5 cells. Cells (100 µL per well, 1 × 10^5^ cells/mL) were seeded into 96-well culture plates. After incubation for 24 h, the medium was replaced with phosphate-buffered saline (PBS) (Gibco, CA, United States) to wash the cells, and then treated with medium supplemented with the different doses of QXHZF-containing serum which was diluted and supplied to 10% by control rat serum. Subsequently, 100 μL per well of 10% (v/v) CCK8 solution (Vazyme, Nanjing, China) was added and cells incubated for 1 h in a 37°C incubator. Finally, absorbance was measured at 450 nm using a microplate reader (BioTek Instruments, VT, United States).

#### 2.1.5 Western blot

Peritoneal tissue samples from mice were ground in a pestle in RIPA lysis buffer (Thermo, MA, United States) supplemented with a 1:50 dilution of protease and phosphatase inhibitors (Beyotime Biotechnology, Shanghai, China) and a 1:1,000 dilution of nuclease solution (Biolonase, Shanghai, China). HMrSV5 cells were seeded into 6-well culture plates at 1 × 10^5^ cells/mL (2 mL per well). Cells were treated as described above. After washing twice with PBS, cells were lysed on ice for 30 min in RIPA lysis buffer and centrifuged (12,000 × g 15 min, 4°C). Supernatants were harvested and the protein concentration measured using a BCA protein assay kit (Thermo, MA, United States). After 10 min protein denaturation at 99°C, samples (20 μg protein per lane) were separated on ExpressPlus™ PAGE Gel (GenScript Corporation, NJ, United States) and transferred to polyvinylidene fluoride membranes. Then, membranes were blocked with 5% BSA in TBST for 1 h at room temperature and incubated with the following primary antibodies overnight: smooth muscle actin (SMA), actin, and estrogen receptor (ER) (Proteintech, Wuhan, China); vimentin, B-cell lymphoma-2 (BCL2), BCL2-Associated X protein (Bax), and p-Raf (CST, MA, United States of America); and rapidly accelerated fibrosarcoma (Raf), mitogen-activated protein kinase (MEK), p-MEK, extracellular regulated protein kinases (ERK), and p-ERK (Santa Cruz, CA, United States). After washing with TBST, membranes were incubated with secondary antibodies (Proteintech, Wuhan, China) for 1 h at room temperature. Images were generated using a chemiluminescence system (GE, Boston, United States) and ImageJ software was used to evaluate gray values in each lane.

#### 2.1.6 Quantitative real-time PCR (qRT-PCR)

HMrSV5 cells were cultured as described, followed by extraction of total RNA using TRIzol reagent (Vazyme, Nanjing, China). After measuring RNA concentration, samples were reverse transcribed to complementary DNA using an All-In-Two RT SuperMix for qPCR Kit (Vazyme, Nanjing, China). qRT-PCR was conducted using the ABI PRISM 7500 PCR system (Applied Biosystems, CA, United States). Primer sequences (Sangon Biotech, Nangjing, China) are listed in Supplementary Table S1. Gene expression levels were calculated using the 2^−△△CT^ method.

#### 2.1.7 TdT-mediated dUTP Nick-End Labeling (TUNEL) staining

HMrSV5 cells were treated as described above, followed by the TUNEL staining using a TUNEL kit (Beyotime Biotechnology, Shanghai, China). In brief, cells washed with PBS and fixing in 1% paraformaldehyde for 30 min. After another wash with PBS, cells were incubated in PBS containing 0.3% Triton X-100 for 5 min at room temperature. TUNEL working solution (50 μL/well) was adding to samples for incubation away from light for 30 min. After washing twice with PBS, cells treated with anti-fluorescence quenching solution were observed under a fluorescence microscope (Leica, Qingdao, China). The TUNEL signal was detected as red fluorescence, using the excitation and emission wavelengths of Cy3 (550 and 570 nm, respectively).

#### 2.1.8 Flow cytometry

HMrSV5 cells were treated as described above and apoptosis assessed using an Annexin V-FITC Apoptosis Detection Kit (Beyotime Biotechnology, Shanghai, China), according to the manufacturer’s instructions. Cells were harvested and resuspended in binding buffer containing annexin V-FITC and propidium iodide (PI). Samples were incubated for 15 min at room temperature protected from light and analyzed by flow cytometry (BD Biosciences, NJ, United States). The maximum excitation wavelength of annexin V-FITC is 488 nm and the emission wavelength is 520 nm. The maximum excitation wavelength of PI is approximately 535 nm and the emission wavelength is 617 nm.

### 2.2 Network pharmacology analysis

#### 2.2.1 Identification of QXHZF compounds and target genes

First, the main active compounds of QXHZF were searched from the Traditional Chinese Medicine Systems Pharmacology (TCMSP, https://tcmspw.com/tcmsp.php) database using the screening criteria: oral availability (OB) ≥ 30% and drug likeness (DL) ≥ 0.18. Compounds were also obtained by literature review; however, they did not meet the screening criteria. Target genes corresponding to active compounds were obtained from the TCMSP and SWISS Target Prediction (http://www.swisstargetprediction.ch/) databases.

#### 2.2.2 Prediction of potential PF-associated targets of QXHZF

The Genecards (https://www.genecards.org/) and OMIM (https://omim.org/) databases were searched using the key word “peritoneal fibrosis” to obtain related genes. The Bioinformatics and Evolutionary Genomics (http://bioinformatics.psb.ugent.be/webtools/Venn/) website was used to calculate and draw custom Venn diagrams between QXHZF target genes and genes related to PF, and intersecting genes were considered as potential targets of QXHZF in PF.

#### 2.2.3 Establishment of a compound-target network

A compound-target network, based on QXHZF compounds and their potential targets, was visualized using Cytoscape version 3.8.2.

#### 2.2.4 Protein-protein interaction (PPI) and key gene analyses

PPI analysis was conducted using the STRING website (https://cn.string-db.org/). The results of PPI analysis were imported into Cytoscape to investigate key targets. Two Cytoscape plugins were used to identify key targets: 1) the Analyze Network tool was applied to obtain the degree of target genes, and 2) the CytoHubba plugin was used to identify the top 20 genes in the PPI network and to establish a critical subnetwork.

#### 2.2.5 Biological function analysis

Gene Ontology (GO) analysis was performed to reveal relevant underlying biological processes (BP), cellular components (CC), and molecular functions (MF). Kyoto Encyclopedia of Genes and Genomes (KEGG) pathway enrichment analysis was used to identify key signaling pathways. Enrichment analysis was implemented using R software version 4.0.3 (Bioconductor, clusterProfiler) with the parameters: adjusted P value < 0.05 and q-value < 0.05. Images were plotted using https://www.bioinformatics.com.cn (last accessed on 10 July 2023).

### 2.3 Serum metabolomics analysis

#### 2.3.1 Metabolite extraction

The LC/MS system for metabolomics analysis consisted of a Waters Acquity I-Class PLUS ultra-high performance liquid tandem Waters Xevo G2-XS QT high resolution mass spectrometer (Waters, MA, United States). The column used was a UPLC HSS T3 column (1.8 μm, 2.1 × 100 mm; Waters, MA, United States). Positive ion mode analysis was conducted using: mobile phases A (0.1% formic acid aqueous solution) and B (0.1% formic acid acetonitrile). Negative ion mode analysis was conducted using: mobile phases A (0.1% formic acid aqueous solution) and B (0.1% formic acid acetonitrile). The gradients of mobile phase A and mobile phase B are presented in Supplementary Table S2. The injection volume was 1 μL.

#### 2.3.2 LC-MS/MS analysis

A Waters Xevo G2-XS QTOF high resolution mass spectrometer was used to collect primary and secondary mass spectrometry data in MSe mode, under the control of MassLynx V4.2 acquisition software (Waters, MA, United States). In each data acquisition cycle, dual-channel data acquisition was performed simultaneously at both low (2 V) and high (10–40 V) collision energies. Scanning frequency was 0.2 s per mass spectrum. ESI ion source parameters were as follows: capillary voltage, 2000 V (positive ion mode) or −1500 V (negative ion mode); cone voltage, 30 V; ion source temperature, 150°C; solvent gas temperature, 500°C; backflush gas flow rate, 50 L/h; solvent gas flow rate, 800 L/h.

#### 2.3.3 Data preprocessing and annotation

Raw data collected using MassLynx V4.2 were processed using Progenesis QI software for peak extraction, peak alignment, and other data processing operations, based on the Progenesis QI software online METLIN database and a self-built biomarker library, for identification; theoretical fragment identification and mass deviation were within 100 ppm.

#### 2.3.4 Metabolomic data analysis

Principal components analysis, partial least-squares discriminant analysis (PLS-DA), orthogonal partial least-squares analysis (OPLS), and Orthogonal Partial Least Squares Discrimination Analysis (OPLS-DA) were performed using BMKCloud (www.biocloud.net). Differential metabolites between the Control vs PD and PD vs PC groups, based on *t*-test (P < 0.05) and VIP value (VIP > 1), were identified according to the online databases: HMDB (http://www.hmdb.ca), ChemSpider (http://www.ch emspider.com), mzCloud (https://www.mzcloud.org/), and KEGG (http://www.kegg.jp).

### 2.4 Integration of serum metabolomics and network pharmacology data

The MetaboAnalyst 3.5 (www.metaboanalyst.ca) modules, joint-pathway analysis and network analysis, were applied for pathway analysis of target genes and differential metabolites to analyze biological mechanisms. The index parameters, P < 0.05 and impact value > 0.1, were used to identify the most relevant pathways.

### 2.5 Molecular docking

Protein crystal structures of the two core targets were obtained from RSCB PDB databases (https://www.rcsb.org/) and the 3D structures of the five main compounds of QXHZF were gained from the PubChem database (https://pubchem.ncbi. nlm.nih.gov). Subsequently, the energy of the structures was minimized by Avogadro software Version 1.2.0 under the MMFF94 force field. The two proteins were subsequently hydrotreated using PyMOL Version 2.5.2. The Pdbqt format files of the two proteins and five small-molecule ligands were converted using the ADFR software Version 1.0. A suitable box was constructed at the center of the protein to fully wrap the protein before molecular docking using AutoDock Vina Version 1.1.2. The docking conformation with the lowest output binding energy was considered the optimal binding conformation. The LigPlot software Version 2.2.4 was used to visualize the 2D interaction diagrams.

### 2.6 Statistical analysis

All experiments were performed independently at least three times. Data are presented as mean ± standard error of the mean (SEM) and were analyzed by *t*-test or one-way ANOVA using SPSS 22.0 statistical software (IBM, Armonk, United States). P < 0.05 was considered statistically significant. Graphs were generated using GraphPad Prism (GraphPad, CA, United States).

## 3 Results

### 3.1 The antifibrosis effect of QXHZF during PD

**FIGURE 2:**
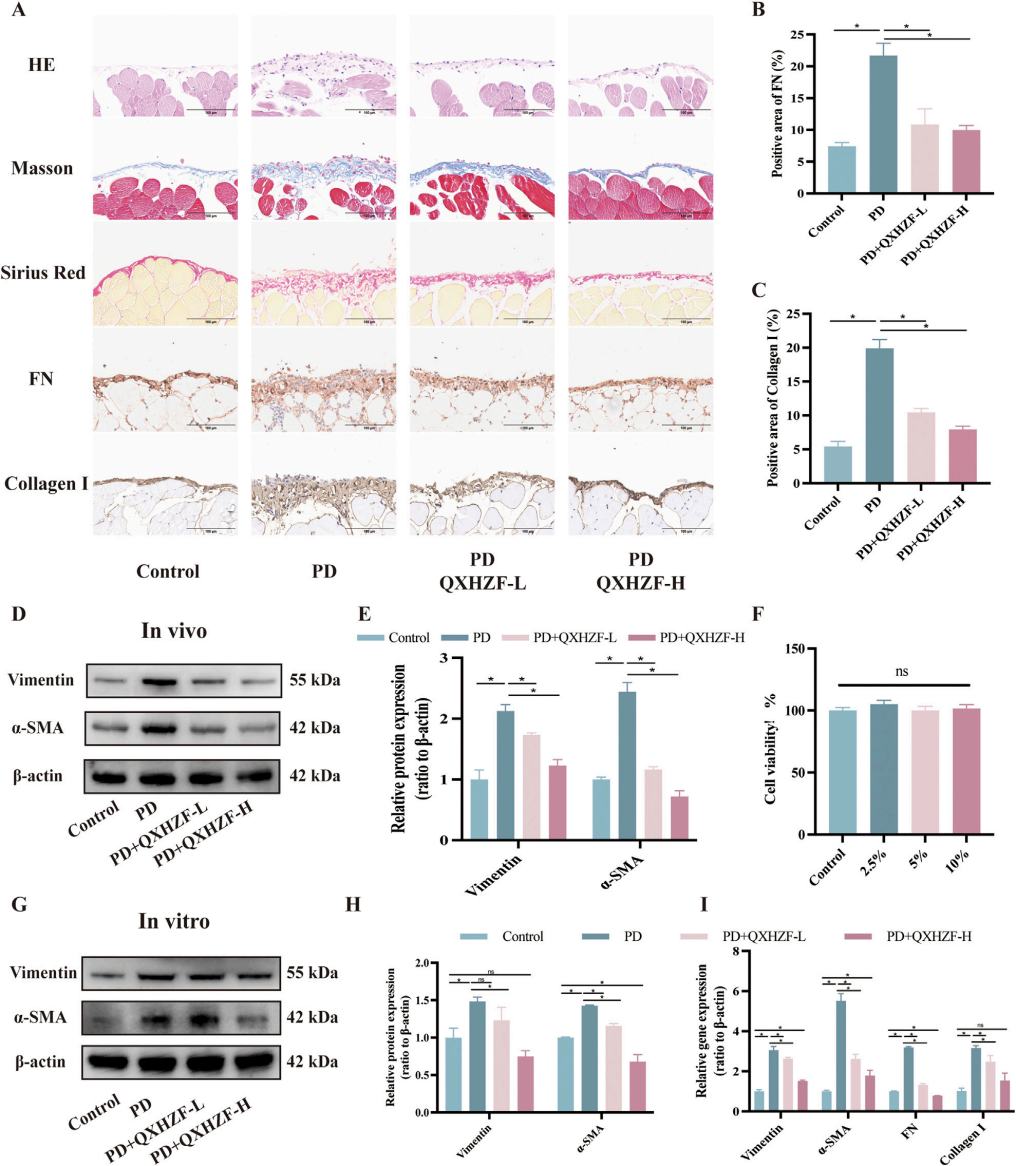
The antifibrosis effect of QXHZF during PD. **(A)** HE, Masson, and Sirius red staining, as well as immunohistochemistry analysis of FN and Collagen I, of mice peritoneum samples showing peritoneal histopathological changes and collage deposition (scale bar = 100 μm). **(B)** Statistical analysis of data in IHC of FN, presented as a histogram. **(C)** Statistical analysis of data in IHC of Collagen I, presented as a histogram. **(D)** Western blot analysis of α-SMA and vimentin expression levels in peritoneal tissue after treatment with 4.25% PDF, with or without QXHZF-L and QXHZF-H gavage. **(E)** Statistical analysis of data in D, presented as a histogram. **(F)** Effect of various doses of QXHZF-containing serum on HMrsv5 cell viability. The different doses of QXHZF-containing serum was diluted and supplied to 10% by control rat serum. **(G)** Western blot analysis of α-SMA and vimentin expression levels in HMrsv5 cells after exposure to 3% PDF, with or without QXHZF-L and QXHZF-H treatment. **(H)** Statistical analysis of data in G, presented as a histogram. **(I)** qRT-PCR assay of α-SMA, vimentin, collagen I, and FN mRNA expression levels in HMrSV5 cells. Data are presented as mean ± SEM of at least three independent experiments (*P < 0.05).

To assess the antifibrosis effect of QXHZF during PD, we conducted a range of *in vivo* and *in vitro* experiments. For animal experiments, mice were administered daily intraperitoneal 4.25% dextrose PDF injections for 28 days to establish a PF model. PM samples were collected from the model mice and their pathological changes assessed by Hematoxylin-Eosin and Masson staining. In addition, we conducted Sirius red and immunohistochemical staining for fibronectin (FN) and Collagen I, to evaluate extracellular matrix (ECM) deposition in peritoneal tissue (Figures 2A–C). In the PD group, cytoplasmic inclusions were detected in the mesothelial layer of the PM, accompanied by sub-mesothelial thickening and ECM deposition, indicating a clear propensity to fibrosis in PD group mice. Moreover, these pathological alterations were mitigated by QXHZF treatment in a dose dependent manner. In addition, the protein expression of MMT markers, such as α-SMA and vimentin, was augmented in the PDF group and dose-dependently reversed by QXHZF (Figures 2D, E). Further, *in vitro* experiments generated consistent results. To explore whether QXHZF-containing serum had toxic effects on HMrSV5 cells, we conducted CCK8 assays, which demonstrating that it exhibited no toxicity. In subsequent experiments, 10% and 5% QXHZF-containing serum was used as high and low doses, respectively (Figure 2F). As shown in Figures 2G, H, levels of α-SMA and vimentin expression were significantly elevated in the PD group, while they were clearly suppressed by high dose QXHZF treatment. Transcription level data generated by PCR reflected a similar tendency at the RNA level. Gene expression levels of α-SMA, Vimentin, Collagen I, and FN were significantly higher in the PD group than those in the Control group, and were dose-dependently reduced by QXHZF administration (Figure 2I). Together, these results demonstrate the antifibrosis effect of QXHZF during PD.

### 3.2 Network pharmacology analysis

**FIGURE 3:**
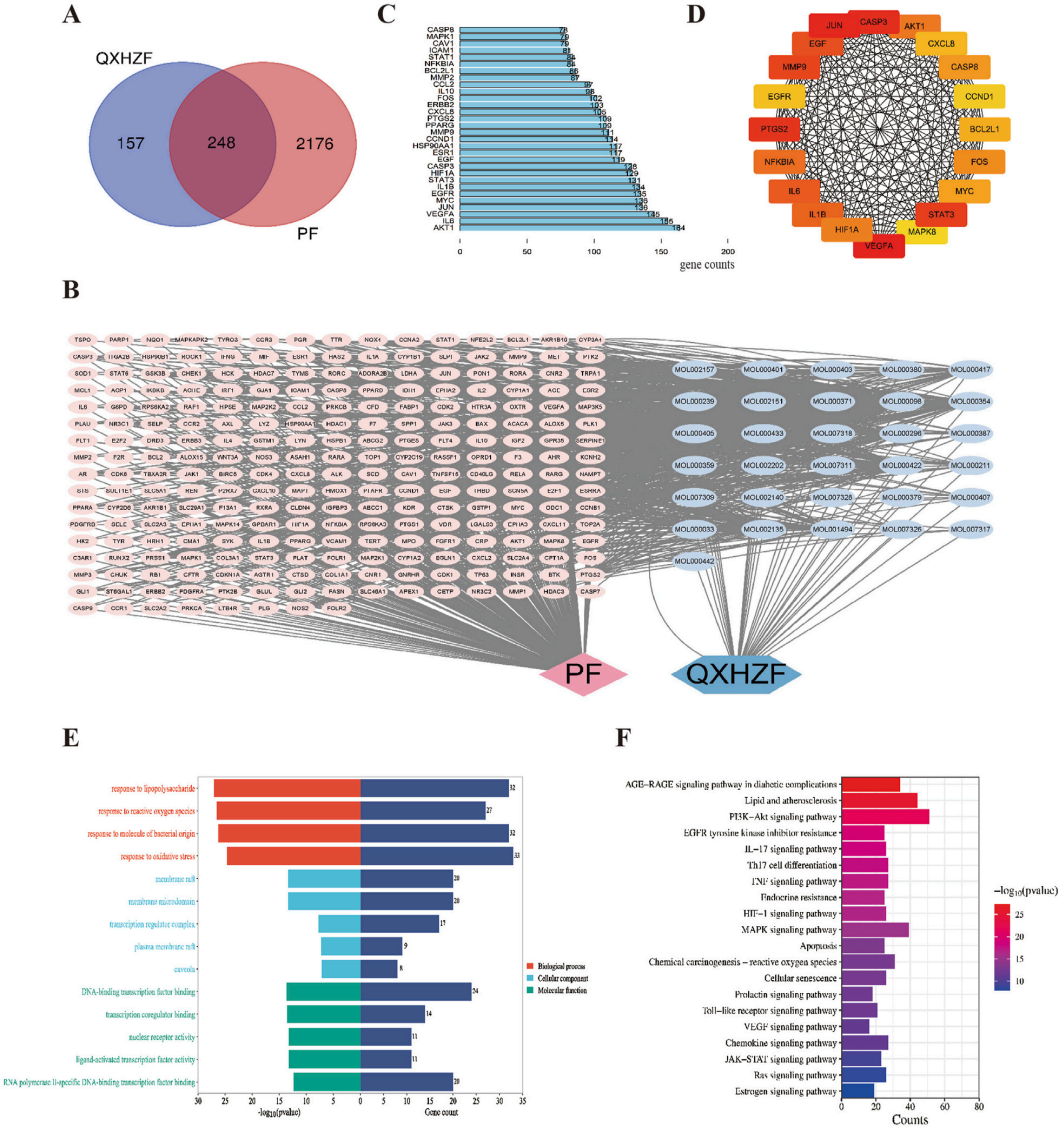
Network pharmacology analysis of the mechanism underlying QXHZF activity in the treatment of PF. **(A)** Venn diagram of QXHZF targets and PF-related genes. **(B)** A QXHZF-PF pharmacology network of compounds and target genes. **(C)** The top 30 genes in the PPI network sorted by degree. **(D)** Key subnetwork detected using CytoHubba. **(E)** Bar chart showing the main BP, CC, and MF terms identified. **(F)** Bar chart showing the main enriched KEGG pathways.

In order to obtain more data to explore deeper mechanisms, we performed network pharmacology. Active compounds in QXHZF were screened from the TCMSP using the criteria, OB ≥ 30% and DL ≥ 0.18, and some active ingredients were identified from recent publications related to PF (Zhu et al., 2022; Xie et al., 2023). A total of 24 active components from Huangqi, eight from Jixuecao, and eight from Chuanxiong were identified; after deduplication, 37 active QXHZF components were obtained. Furthermore, 405 targets of QXHZF were obtained from the TCMSP and Swiss Target Prediction databases, based on the retrieved components. Taking the intersection of these targets with 2,424 disease targets identified from Genecard and OMIM using the search term “peritoneal fibrosis” as a keyword, 248 potential targets of QXHZF related to PF were identified (Figure 3A). To further investigate the mechanisms of QXHZF activity in the context of PF, a compounds-targets network was constructed using Cytoscape, including 37 active components and 248 potential targets (Figure 3B). The top five components sorted by degree (i.e., linkage to more targets) were quercetin, kaempferol, isorhamnetin, myricanone, and jaranol, which are likely key active constituents of QXHZF in the context of PF (Supplementary Table S3). A PPI network, including 248 potential targets, was constructed using the STRING database and imported into Cytoscape for core gene analysis. Core genes were identified by two methods: 1) The top 30 genes in the PPI network were selected by sorting according to degree (Figure 3C); and 2) Key subnetworks consisting of 20 genes were detected using CytoHubba (Figure 3D). Combination of the results of these two methods identified core genes including estrogen receptor 1 (ESR1) and BCL2-like protein 1 (BCL2L1).

The potential mechanisms underlying the effects of QXHZF were predicted by GO and KEGG pathways enrichment analyses. The top 5 enriched GO analysis terms, ranked by P value, are presented in Figure 3E. In detail, BP related to QXHZF included: chemical stress (GO:0062197), lipopolysaccharide (GO:0032496), reactive oxygen species (GO:0000302), molecule of bacterial origin (GO:0002237), and oxidative stress (GO:0006979). Regarding CC, terms identified were: membrane raft (GO:0045121), membrane microdomain (GO:0098857), transcription regulator complex (GO:0005667), plasma membrane raft (GO:0044853), and caveola (GO:0005901). Enriched MF terms were: DNA-binding transcription factor binding (GO:0140297), transcription coregulator binding (GO:0001221), nuclear receptor activity (GO:0004879), ligand-activated transcription factor activity (GO:0098531), and RNA polymerase II-specific DNA-binding transcription factor binding (GO:0061629). Furthermore, KEGG analysis of QXHZF identified apoptosis (hsa04210), estrogen signaling pathway (hsa04915), and Ras signaling pathway (hsa04014) as enriched (Figure 3F).

### 3.3 Serum metabolomics analysis

**FIGURE 4:**
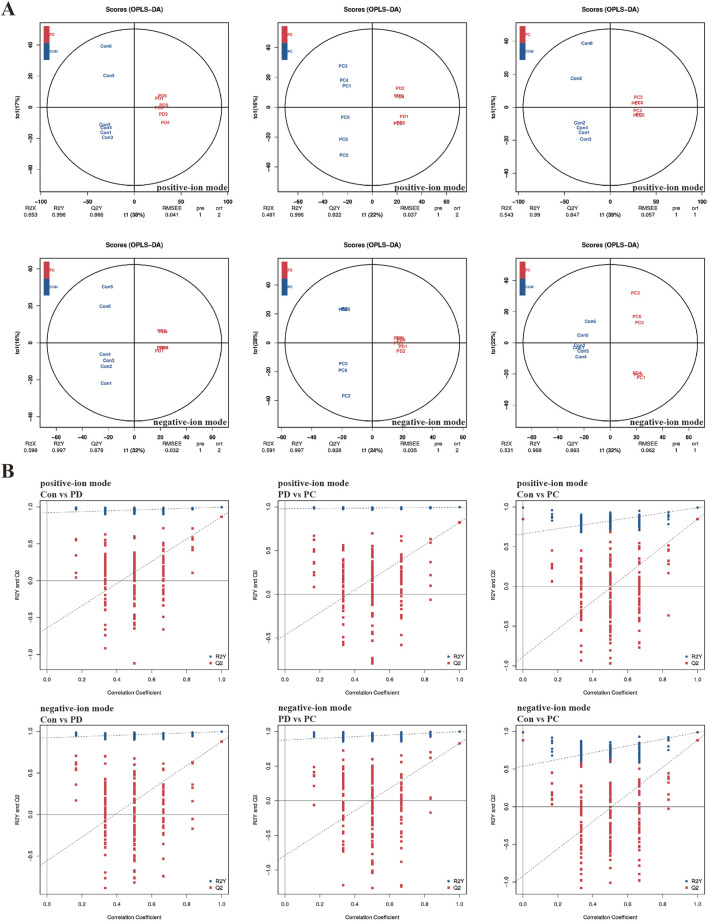
Multivariate analysis of LC-MS/MS data. **(A)** OPLS-DA score plots of Con, PD, and PC in positive-ion mode and negative-ion mode. **(B)** Permutation verification analysis of Con, PD, and PC in positive-ion mode and negative-ion mode. Con, control; PD, peritoneal dialysis; PC, PD + QXHZF.

**FIGURE 5:**
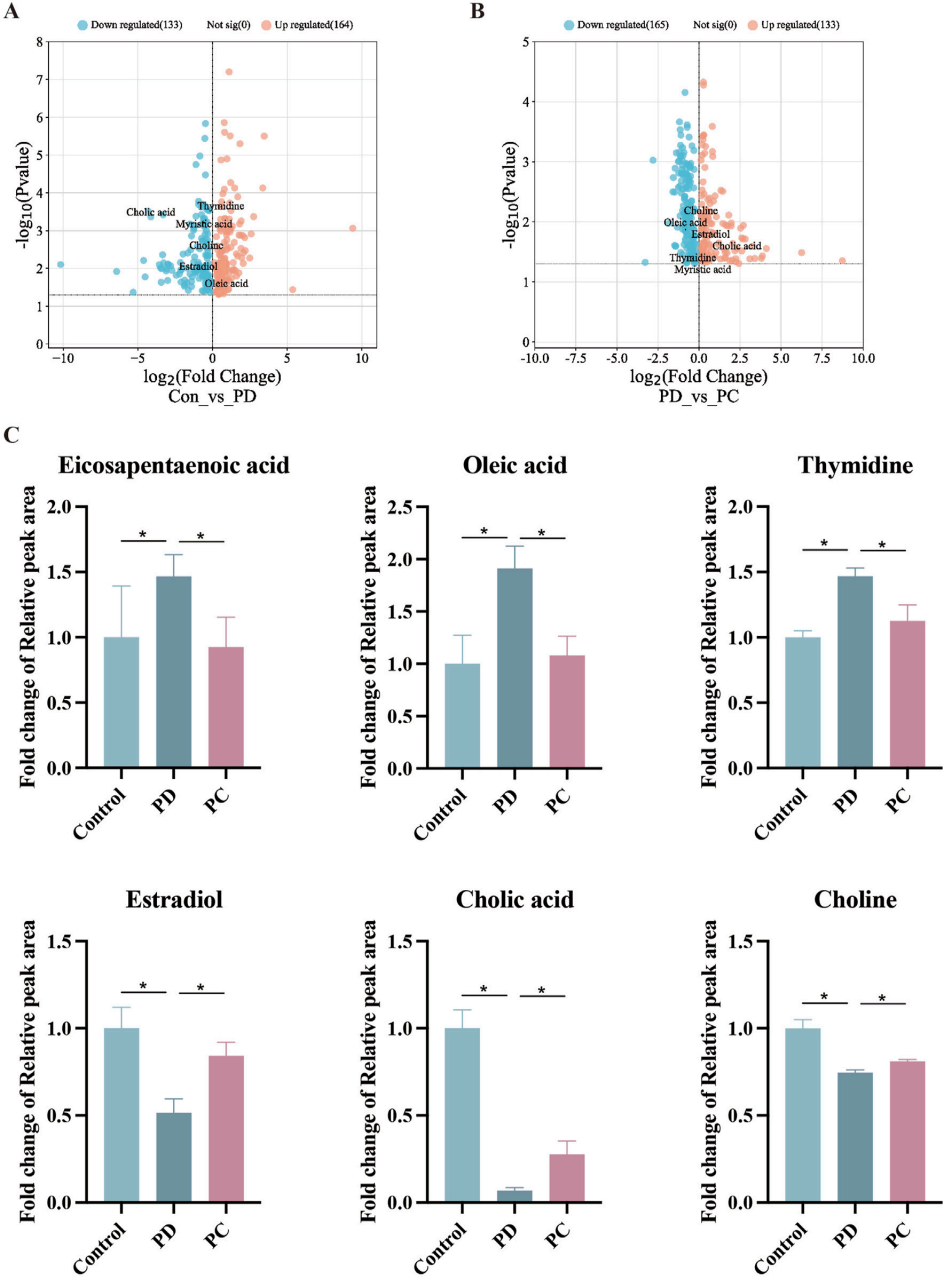
Comparison of relative peak areas of differential metabolites identified by serum metabolomics analysis. **(A)** Volcano map of differential metabolites between Con and PD. **(B)** Volcano map of differential metabolites between PD and PC. **(C)** Fold change of the relative peak area in the date of potential metabolites in serum metabolomics. Data represent mean ± SEM (n = 6), *P < 0.05. Con, control; PD, peritoneal dialysis; PC, PD + QXHZF.

To dig out underlying mechanisms in metabolism, we preformed the serum metabolomics analysis. Significant separation between each pair of groups was observed in OPLS-DA score plots (Figure 4A). Permutation verification analysis was used to assess the reliability of the OPLS-DA model (Figure 4B). The OPLS-DA model was effective for prediction, according to R2Y and Q2 values, which were close to 1, suggesting that VIP could be used to screen for differential metabolites among the three groups. Therefore, metabolites with P < 0.05 and VIP >1 were screened. In positive ion mode, 1,234 (184 upregulated and 1,050 down-regulated) and 595 (189 up-regulated and 406 down-regulated) differential metabolites were identified between the Control and PD groups and the PD and PC groups, respectively. We next sought to identify metabolites whose levels were reversed in the PD model after treatment with QXHZF. Ninety-six differential metabolites were obtained after intersecting those downregulated in the Control vs. PD in comparison with those upregulated in PD vs. PC groups. Similarly, 56 differential metabolites were obtained from the intersection of those upregulated in Control vs. PD and down-regulated in PD vs. PC (Supplementary Figures S1A, B). Thus, 152 differential metabolites were detected in positive ion mode, consistent with our expectation. Moreover, 150 metabolites were obtained from negative ion mode data (Supplementary Figures S1C, 1D). In total, 299 differential metabolites were collected from both positive and negative ion modes after deduplication (Supplementary Figure S1E), and are presented as volcano maps in Figures 5A, B. The only pathway identified as significant by pathway analysis among these differential metabolites was pyrimidine metabolism (Supplementary Figure S2). Potentially important metabolites were identified by comparing the relative content alteration among differential metabolites. Compared with the Control group, levels of eicosapentaenoic acid, oleic acid, and thymidine were increased, while those of estradiol, cholic acid, and choline were decreased in the PD group (Figure 5C). Notably, serum content of these metabolites was reversed after treatment with QXHZF, returning levels to those comparable with the Control group.

### 3.4 Integration of results from network pharmacology and serum metabolomics analyses

**FIGURE 6:**
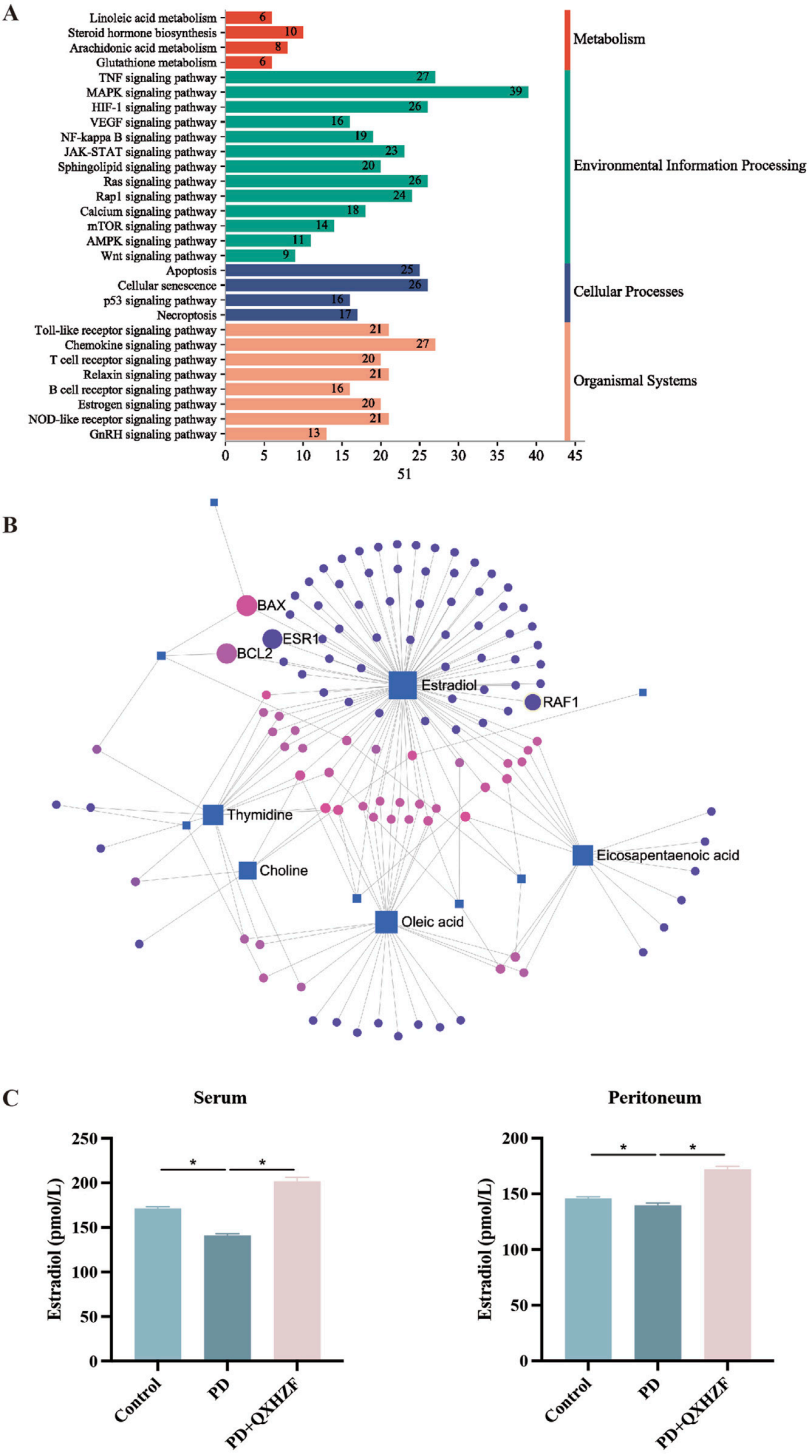
Integrated network pharmacology and serum metabolomics analysis of QXHZF in the treatment of PF. **(A)** Bar chart showing the main terms enriched in KEGG pathways analysis. **(B)** Network of differential metabolites and target genes. **(C)** ELISA assay of estradiol in mouse serum and PM after treatment with 4.25% PDF, with or without QXHZF(High dose) gavage. Data are presented as mean ± SEM of at least three independent experiments (*P < 0.05).

**FIGURE 7:**
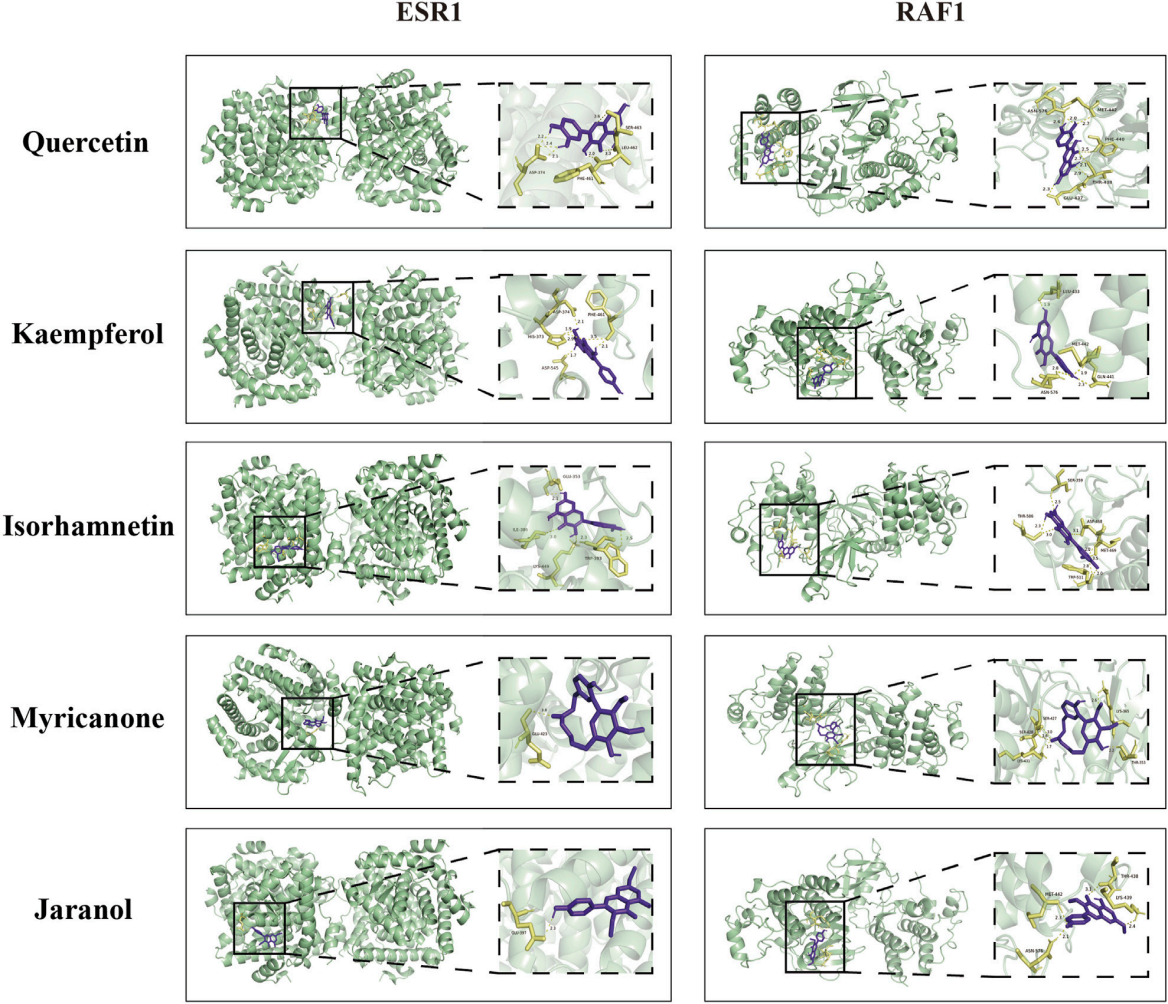
Molecular docking of quercetin, kaempferol, isorhamnetin, myricanone, and jaranol to ESR1 and RAF1.

To comprehensively explore the potential mechanisms underlying the effects of QXHZF on PF, we focused on correlations between the 299 differential metabolites and 248 target genes, to integrate the results of the network pharmacology and metabolomics analyses. Joint pathway analysis was performed with MetaboAnalyst using threshold values of P < 0.05 and impact > 0.1. Steroid hormone biosynthesis in metabolism, Ras signaling pathway in environmental information processing, apoptosis in cellular processes, and estrogen signaling pathway in organismal systems were each enriched for both target genes from network pharmacology and differential metabolites from serum metabolomics analyses (Figure 6A). In addition, we constructed a network among metabolites and target genes to visually explore their relationships (Figure 6B). Estradiol clearly had the strongest correlations with target genes in the network, including ESR1, RAF1, BCL2, and BAX. As estradiol was identified as the core metabolite, we conducted secondary identification of this metabolite in mouse serum and PM by ELISA (Figure 6C). Levels of estradiol in the PD group were less than those in Controls, and intervention with QXHZF significantly reversed this effect, consist with our serum metabolomics results. Combined with the results of compounds-targets network analysis, a molecular docking experiment was performed using screened compounds with the top 5° values and target genes obtained by integrated network analysis, including ESR1 (PDB ID: 3os8) and RAF1 (PDB ID: 3omv). The binding energies of ESR1 to the potential compounds, quercetin, kaempferol, isorhamnetin, myricanone, and jaranol, were −5.17, −5.64, −5.69, −5.58, and −5.3 kcal/mol, respectively, while those of RAF1 with the same compounds were −5.97, −6.55, −6.4, −6.01, and −6.65 kcal/mol, respectively. Hence, all binding energies were < −5 kcal/mol, indicating that the potential compounds have strong affinity for ESR1 and RAF1; the relevant binding conformations are presented in Figure 7 and Supplementary Table S4.

### 3.5 Regulation of apoptosis, estrogen, and Raf/MEK/ERK signaling by QXHZF during PD

**FIGURE 8:**
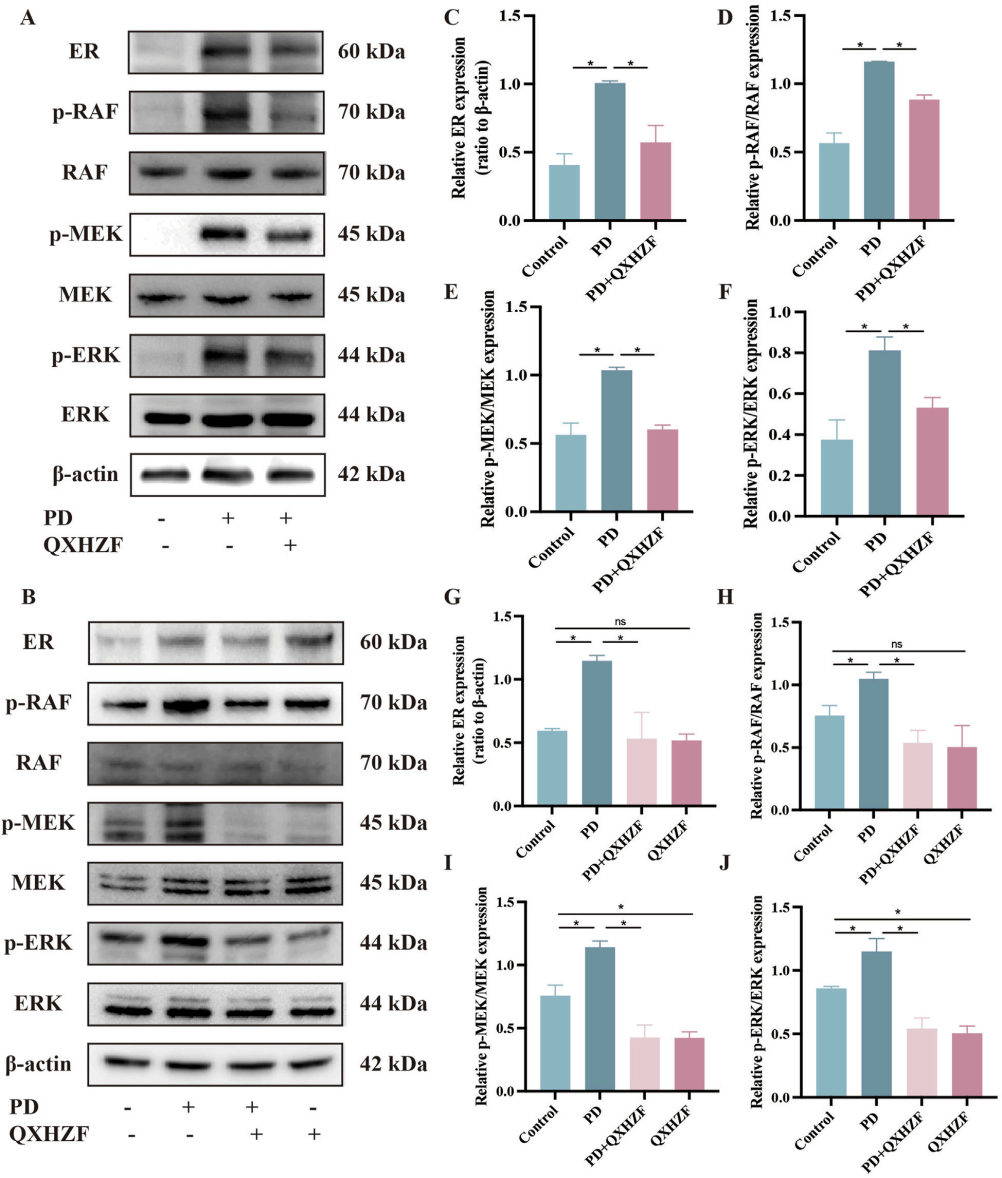
Estrogen and Raf/MEK/ERK signaling participate in QXHZF-mediated regulation of PD-induced PF *in vivo* and *in vitro*. The expression of ER, p-RAF, RAF, p-MEK, MEK, p-ERK, and ERK were assessed by Western blot in peritoneal tissue **(A, C–F)** and HMrSV5 cells **(B, G–J)** after PDF and QXHZF treatment. Data are presented as mean ± SEM of at least three independent experiments (*P < 0.05).

**FIGURE 9:**
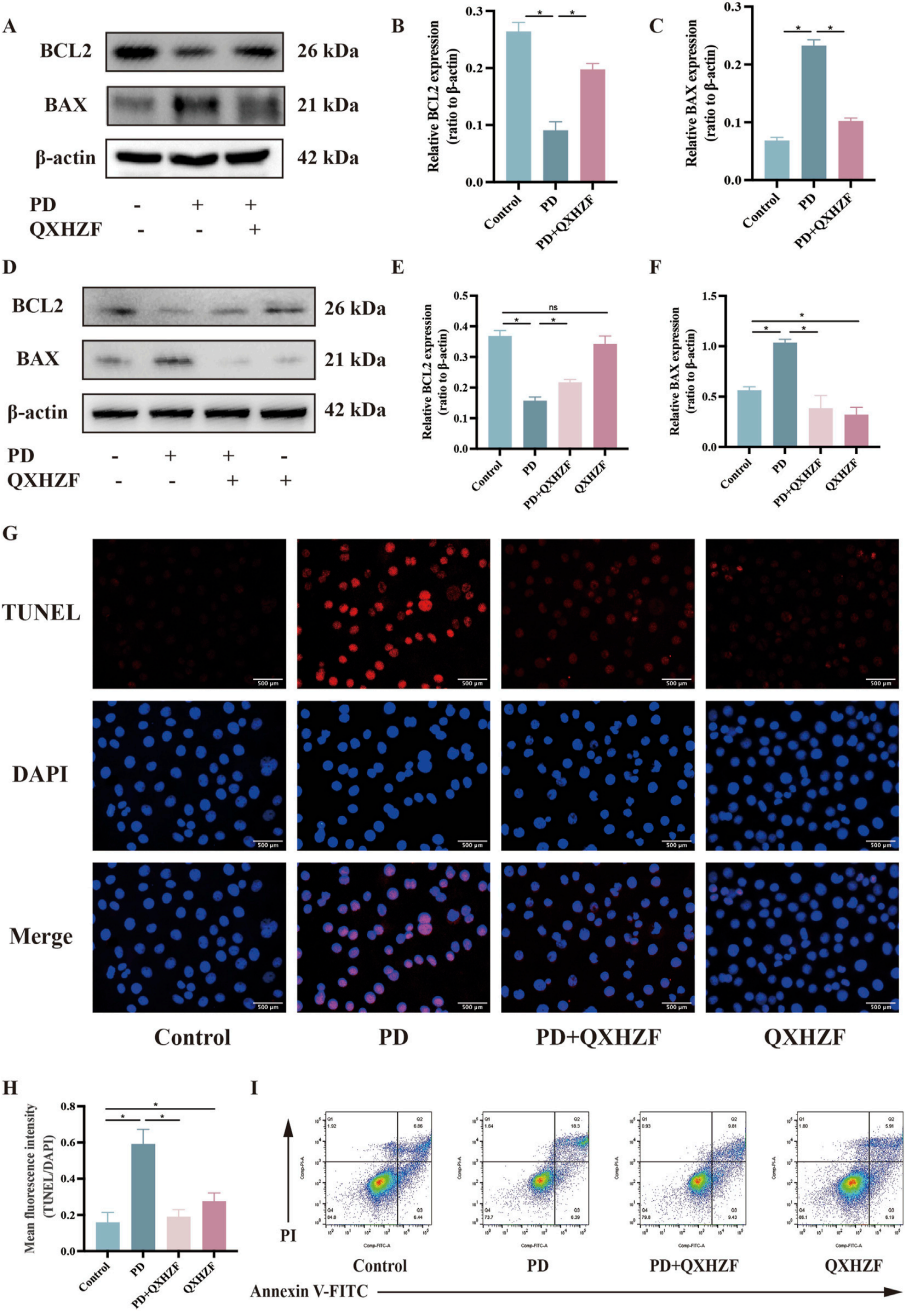
Apoptosis participates in QXHZF-mediated regulation of PD-induced PF *in vivo* and *in vitro*. **(A–F)** BCL2 and BAX expression assessed by Western blot in peritoneal tissue **(A–C)** and HMrSV5 cells **(D–F)** after PDF and QXHZF treatment. **(G–H)** Effect of QXHZF on apoptosis of HMrSV5 cells with PD-induced PF detected by TUNEL staining. **(I)** Effect of QXHZF on apoptosis of HMrSV5 cells with PD-induced PF detected using an Annexin V-FITC apoptosis detection kit. Data are presented as mean ± SEM of at least three independent experiments (*P < 0.05).

Integrated analysis of network pharmacology and serum metabolomics data indicated that QXHZF potentially regulates PF through the estrogen and Ras signaling pathways, as well as *via* apoptosis. Thus, we examined the relevant targets *in vivo* and *in vitro*, to verify the molecular mechanisms involved. Estrogen binds to ER within cells to initiate signaling pathways and influence biological functions (Prossnitz and Barton, 2023). Detection of ER revealed higher expression in the PD group than that in the Control group, which was reduced by treatment with QXHZF (Figures 8A–C, G). Raf, MEK, and ERK are the main targets in the Ras signaling pathway. PDF induced p-Raf/Raf, p-MEK/MEK, and p-ERK/ERK upregulation both *in vivo* and *in vitro*, while QXHZF inhibited the elevated those key markers in Ras signaling driven by PDF (Figures 8A, B, D–F, H–J). These results confirm that the antifibrosis effects of QXHZF during PD is associated with modulation of apoptosis, estrogen, and Ras signaling. Moreover, BCL2 protein levels were downregulated, while those of BAX were upregulated following PDF intervention, and both changes were reversed in response to QXHZF (Figures 9A–F). Further, TUNEL staining (Figures 9G, H) demonstrated apoptosis regulation in response to PDF and QXHZF. The results of annexin V-FITC(+) analysis by flow cytometry (Figure 9I) were consistent with these findings, although statistical analysis revealed a trend toward suppression between the PD and PD + QXHZF groups, but the difference was not statistically significant (Supplementary Figure S3).

## 4 Discussion

In clinical application, QXHZF can effectively improve the symptoms and quality of life of patients undergoing PD. To our best knowledge, this is the first systematic investigation of the mechanism underlying the effect of QXHZF against PF using serum metabolomics combined with network pharmacology analyses. In this study, we confirmed the significant role of QXHZF as a PF suppressor, both *in vivo* and *in vitro*. Subsequently, we implemented network pharmacology analysis to predict the potential mechanisms underlying the effects of QXHZF on PF related to PD. Further, we applied serum metabolomics to identify differential metabolites induced by QXHZF in the context of PF related to PD. Moreover, integrated analysis of network pharmacology and serum metabolomics data identified crucial pathways, metabolites, and targets, which have been mainly enriched in estrogen, apoptosis, and Ras signaling. Finally, we carried out extensive investigations to assess these predicted mechanisms, including molecular docking and experimental verification.

The primary reason for PD failure is the development of PF induced by long-term use of PDF, and there are currently no definitive curative interventions; however, several groups have made efforts to explore approaches to improve PF through different mechanisms. Shinkai et al. found that a selective peroxisome proliferator-activated receptor α (PPARα) modulator ameliorated peritoneal inflammation and fibrosis by stabilizing the nuclear factor-κB (NF-κB) inhibitory protein, IkBα, to inhibit proinflammatory cytokine induction by NF-κB in human peritoneal mesothelial cells, and activating the NLR family pyrin domain containing 3/caspase-1 axis in interferon-γ-induced THP-1 cells (Shinkai et al., 2024). Further, myeloid differentiation factor 2 (MD2) contributes to isoproterenol-induced cardiac inflammation and injuries via the β1-adrenergic receptor (β1-AR)-cAMP-PKA-ROS signaling axis in cardiomyocytes and the β2-AR-cAMP-PKA-ROS axis in macrophages (Qian et al., 2024). A clinical study demonstrated that the administration of low glucose degradation product PDF led to less severe PM damage, in terms of peritoneal microvessel density and peritoneal inflammation, after kidney transplantation (Zhang et al., 2023). Inhibitors of p38 MAPK have an inhibitory effect on PF in mice, and Ikushima et al. confirmed their role in macrophages contributing to PF (Ikushima et al., 2024). Moreover, canagliflozin, a sodium-glucose cotransporter type 2 (SGLT2) inhibitor, mitigates PF and peritoneal function by improving hypoxia and suppressing the HIF-1α/TGF-β/p-Smad3 signaling pathway (Wang J. et al., 2023).

Further, there is increasing evidence that various TCM preparations, or their active ingredients, have anti-fibrosis activities, providing a robust basis for treatment of PF using TCM. The mitophagy activator, salidroside, rescues peritoneal fibrotic responses and cellular senescence induced by Brahma-related gene 1 (BRG1) (Li et al., 2024), which are ascribed to mitochondrial dysfunction and mitophagy inhibition. Mechanistically, BRG1 was recruited to the oxidation resistance 1 (OXR1) promoter to inhibit OXR1 transcription by interacting with forkhead box protein p2. Inhibition of OXR1 abolished the enhancement of BRG1 deficiency in mitophagy, senescence and fibrosis. Salvia miltiorrhiza and its major active component, salvianolic acid A, have beneficial effects in PF, hindering GSK3β hyperactivity to suppress NF-κB signaling and activate Nrf2 signaling (Zhou et al., 2022). Furthermore, baicalein (5,6,7-trihydroxyflavone) has an anti-PF role through modulation of cell proliferation, inflammatory response, and the AGE-RAGE signaling pathway (Lu et al., 2023). Another study revealed that genistein inhibits O-linked-N-acetylglucosaminylation (O-GlcNAcylation) status of hypoxia-inducible factor 1-alpha (HIF-1α) through the mTOR/O-GlcNAc transferase pathway, elevating its ubiquitination and subsequent proteasomal degradation, thereby improving PF (Wang F. et al., 2024).

QXHZF is composed of *A. membranaceus* (Chinese name: Huangqi), *C. asiatica* (Chinese name: Jixuecao), and *L. sinense* (Chinese name: Chuanxiong), which act together to induce qi invigoration, detoxification, and blood activation in the clinic. The major compounds in QXHZF are reported to ameliorate PF through different mechanisms. There are previous reports that its active components, such as astragaloside IV (AS-IV) and Astragalus polysaccharide (APS), have certain efficacy in improving PD-induced PF, and that the underlying mechanism is related to anti-inflammatory responses, suppression of oxidative stress, and modulation of apoptosis, among other processes, involving various signaling pathways, such as the TGF-β, Wnt, and AKT pathways (Huang et al., 2024). AS-IV is reported to effectively improve PF through the PGC-1α/ROS/apoptosis signaling pathway (Xie et al., 2023). In our previous studies, we found that *A. membranaceus* and calycosin ameliorate PF and related muscle atrophy *via* the co-target AR, and modulated the AR/TGF-β1/smads pathway (Sheng et al., 2024); AS-IV has been proven to have a therapeutic effect towards PF (Shan et al., 2024), and attenuates macrophage-derived exosome-induced PF during PD *via* the miR-204–5p/Foxc1 pathway; APS augments the targeted homing of bone marrow mesenchymal stromal cells to peritoneal tissue by modulating pathways downstream of the SDF-1/CXCR4 axis and suppressing MMT, as well as PF(Wang J. et al., 2024); AS-IV effectively promotes the upregulation of Smad7 in the TGF-β1/Smad signaling pathway during HMrSV5 cell MMT to modulate PF (Zhang et al., 2015). Asiaticoside (ASI) is an important active ingredient of *C. asiatica* that inhibits TGF-β1-induced MMT and reactive oxygen species *via* Nrf2 activation, thus protecting the peritoneal membrane and preventing PF (Zhao et al., 2020); ASI regulate the JAK2/STAT3 signaling pathway to inhibit PMCs MMT and alleviate PF (Sun et al., 2023). A previous study of L. sinense revealed that ligustrazine can mitigate LPS-induced apoptosis and fibrosis through inhibition of oxidative stress and p38/MAPK and ROS/MMP-9 activation in PMCs (Zhang et al., 2016). Further, in our previous study, we demonstrated that tetramethylpyrazine ameliorates angiogenesis, PF, and PM injury by inhibiting VEGF/Hippo/YAP signaling (Zhu et al., 2021). Together, the findings of these studies strongly support the anti-PF properties of QXHZF. In this study, we addressed the question of the potential mechanisms underlying the anti-PF effects of QXHZF.

First, we confirmed the anti-PF effect of QXHZF using various experimental methods, including WB, qRT-PCR, and examination of pathological sections. The results of network pharmacological analysis indicated that multiple components of QXHZF exert therapeutic effects on PF related to PD, by modulating numerous targets and pathways. As shown in the compounds-targets network, the top three components of QXHZF, quercetin (Wang Q. et al., 2023), kaempferol (Guan et al., 2023), and isorhamnetin (Zheng et al., 2019), were previously demonstrated to have potential for the treatment of fibrosis, confirming the therapeutic efficacy of QXHZF against this condition. Additionally, ESR1 and BCL2L1 were identified as core genes in the context of QXHZF treatment of PF. ESR1 is a transcription factor that controls gene expression, and its inhibitor, tamoxifen, is reported to strongly influence PF(Loureiro et al., 2013). Further, there is evidence that PDF-induced MMT of PMCs occurs via activation of ESR1 (Zhao et al., 2023). In addition, PMC apoptosis is a major cause of MMT and PF. The BCL2 family of proteins are critical regulators of apoptosis and fibrosis, and BCL2 and BAX commonly serve as apoptosis biomarkers. Similarly, apoptosis, the estrogen signaling pathway, and Ras signaling were identified by pathway enrichment analysis. There is evidence that suppressing Ras/RAF/MEK/ERK signaling activation can delay fibrosis progression (Yu et al., 2022, Chen et al., 2023). In summary, our data indicate that various components of QXHZF may act on PF through multiple pathways.

During PD, there is a significant increase in nutritional supplementation, accompanied by the rise of cytokine production and peritoneal injury (Si et al., 2019). The mechanism of cellular metabolism caused by PD, as well as the drugs targeted it for the treatment of PD-associated PF, has not been well studied. Thus, we performed the serum metabolomics analysis to dig out underlying mechanisms in metabolism. In our serum metabolomics analysis, 299 differential metabolites were collected from both positive and negative ion modes after deduplication. Pyrimidine metabolism was identified as significant by pathway analysis. The protection against liver fibrosis conferred by the hydroxysteroid, 17-beta dehydrogenase 13 (HSD17B13), is associated with reduced dihydropyrimidine dehydrogenase-mediated pyrimidine catabolism (Luukkonen et al., 2023). Moreover, a series of tetrahydropyrido [4,3-d]pyrimidine derivatives demonstrated excellent activity against cardiac and hepatic fibrosis (Jiang et al., 2020). These pieces of evidence strongly support the argument for the role of pyrimidine metabolism in modulating fibrosis. Our findings are the first to identify the relationship between pyrimidine metabolism and PF, and in-depth research on the relevant mechanisms is warranted in the future.

The major differential metabolites identified among the Control, PF, and QXHZF groups were eicosapentaenoic acid, oleic acid, thymidine, estradiol, cholic acid, and choline, among others. Combining these differential metabolites and potential targets by joint pathway analysis suggested the involvement of steroid hormone biosynthesis in metabolism, Ras signaling in environmental information processing, apoptosis in cellular processes, and estrogen signaling in organismal systems, which is generally consistent with the results of KEGG analysis of network pharmacology data. Importantly, estradiol is an estrogen, which is associated with several physiological and pathological processes, including fibrosis. A clinical study showed that postmenopausal women with nonalcoholic steatohepatitis had a higher risk of liver fibrosis than premenopausal women (Klair et al., 2016). Further, animal research demonstrated estrogen deficiency thioacetamide-induced oxidative damage, inflammation, and fibrosis in the liver (Lee et al., 2019). Furthermore, estrogen signaling contributes to lung fibrosis (Sathish et al., 2015; Tzouvelekis and Bouros, 2019). Similarly, our findings indicated reduced estradiol in the PD group, consistent with the clinical manifestation of a decrease in systemic hormone levels in patients with PD, and this was reversed by treatment with QXHZF. Further network analysis indicated that estradiol was most strongly correlated with target genes, including ESR1, RAF1, BCL2, and BAX, while the potential compounds screened as the top 5, according to degree in the compounds-targets network, had strong affinity for ESR1 and RAF1, supported by the results of our molecular docking analysis. To further investigate the above predictions, we conducted verification experiments by WB of *in vivo* and *in vitro* samples. Markers of apoptosis (BCL2 and BAX), estrogen signaling (ER), and Ras signaling (Raf, MEK, and ERK) were activated in the PDF-induced group, as evident from the lower expression of BAX and higher expression of BCL2, ER, p-Raf/Raf, p-MEK/MEK, and p-ERK/ERK, while treatment with QXHZF reversed these changes in signaling molecules, ultimately mediating inhibition of PDF-induced PF. The ER signaling pathway modulates the gene expression of target tissues, resulting in rapid changes in the cellular events. However, recent studies suggest that Ras signaling pathway (Hong and Choi, 2018) and apoptosis (Lewis-Wambi and Jordan, 2009) are regulated by estrogen in several tissues. Ras signaling, and specifically Raf-1, a downstream molecule in this pathway, has crucial anti-apoptosis effects (Ma et al., 2017). According to a previous study, a specific drug suppressed Raf/MEK/ERK pathway to ameliorate acute inflammation and activated Akt signaling to promote cell survival via inhibiting apoptosis (Man et al., 2005). The Akt pathway was also enriched in our pathway analyses; hence, further exploration of how Ras signaling and apoptosis interact in QXHZF-mediated modulation of PF is warranted.

Notably, although the upregulation of ER detected in our study could potentially be attributed to feedback arising from estradiol deficiency, which was revealed by serum metabolomics analysis, it is also possible that the variance observed is due to analysis by serum metabolomics, rather than tissue metabolomics. More in-depth research to reveal the complex related mechanisms will be essential.

At the beginning of the project, we followed the protocol used in previous investigations using 6–8-week-old of male C57BL/6J mice. Hence, the serum samples used in metabolomics analysis were all from male mice; however, on analysis of our data, we unexpectedly identified significant differences and relevance of the estrogen signaling pathway in serum and peritoneal tissues. We consider that these findings reflect the fact that estradiol and estrogen receptors are expressed in male, as well as female, mice. This result will inform further research on the relevance of sex differences in this pathway.

## 5 Conclusion

Our data highlight apoptosis, estrogen signaling, and Ras signaling as the primary pathways influenced by QXHZF in treatment of PF related to PD. Serum metabolomics and network pharmacology analyses indicate that QXHZF likely contributes to PF through multiple targets and pathways; the detailed mechanisms will be investigated in greater depth in future studies.

## Data Availability

The datasets presented in this study can be found in online repositories. The names of the repository/repositories and accession number(s) can be found in the article/Supplementary Material.
