# Supplementary figures and images for: Integrated serum metabolomics and network pharmacology reveal molecular mechanism of Qixue Huazheng formula on peritoneal fibrosis
Source: Front Pharmacol. 2025 Jan 23;16:1515038. doi: 10.3389/fphar.2025.1515038 (PMC11799242; doi:10.3389/fphar.2025.1515038)

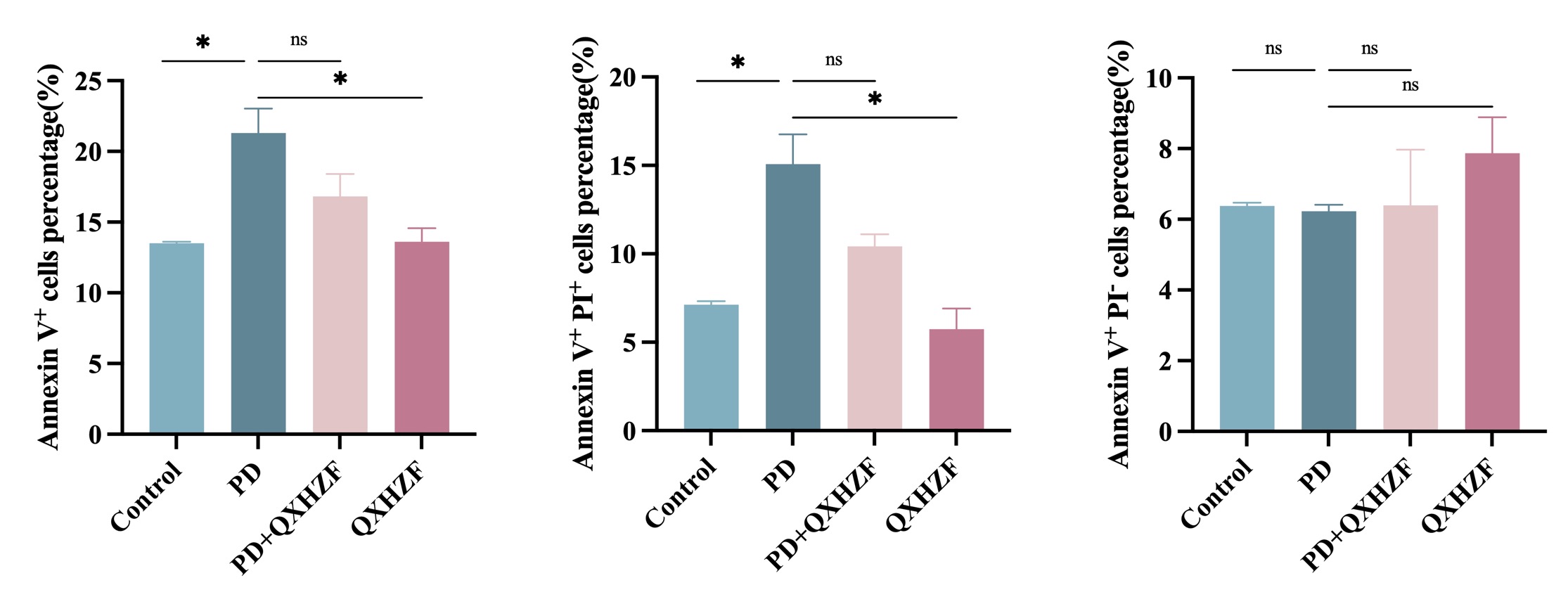

Supplement: Supplementary file 1 [file Image3.jpeg]

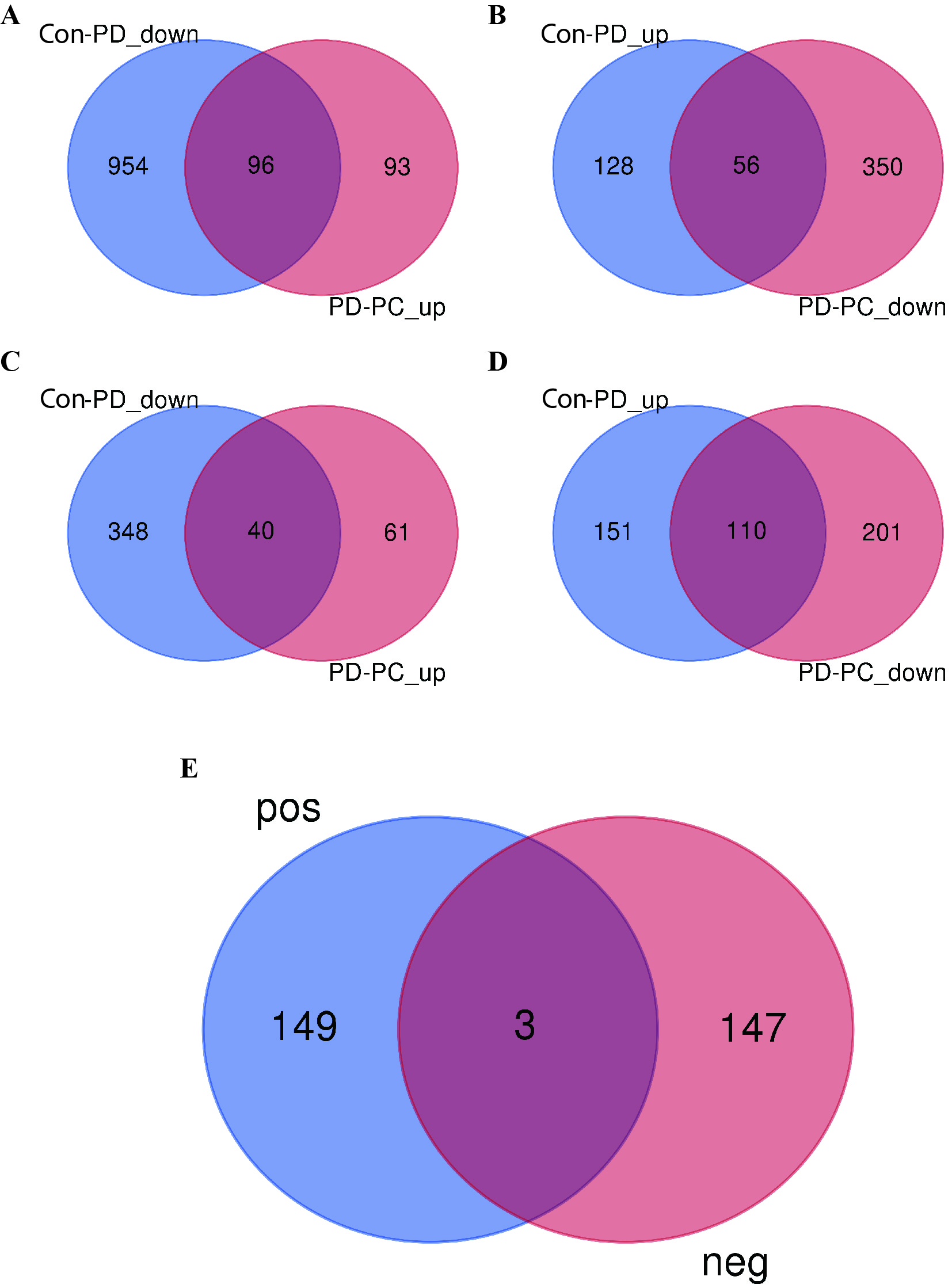

Supplement: Supplementary file 3 [file Image1.jpeg]

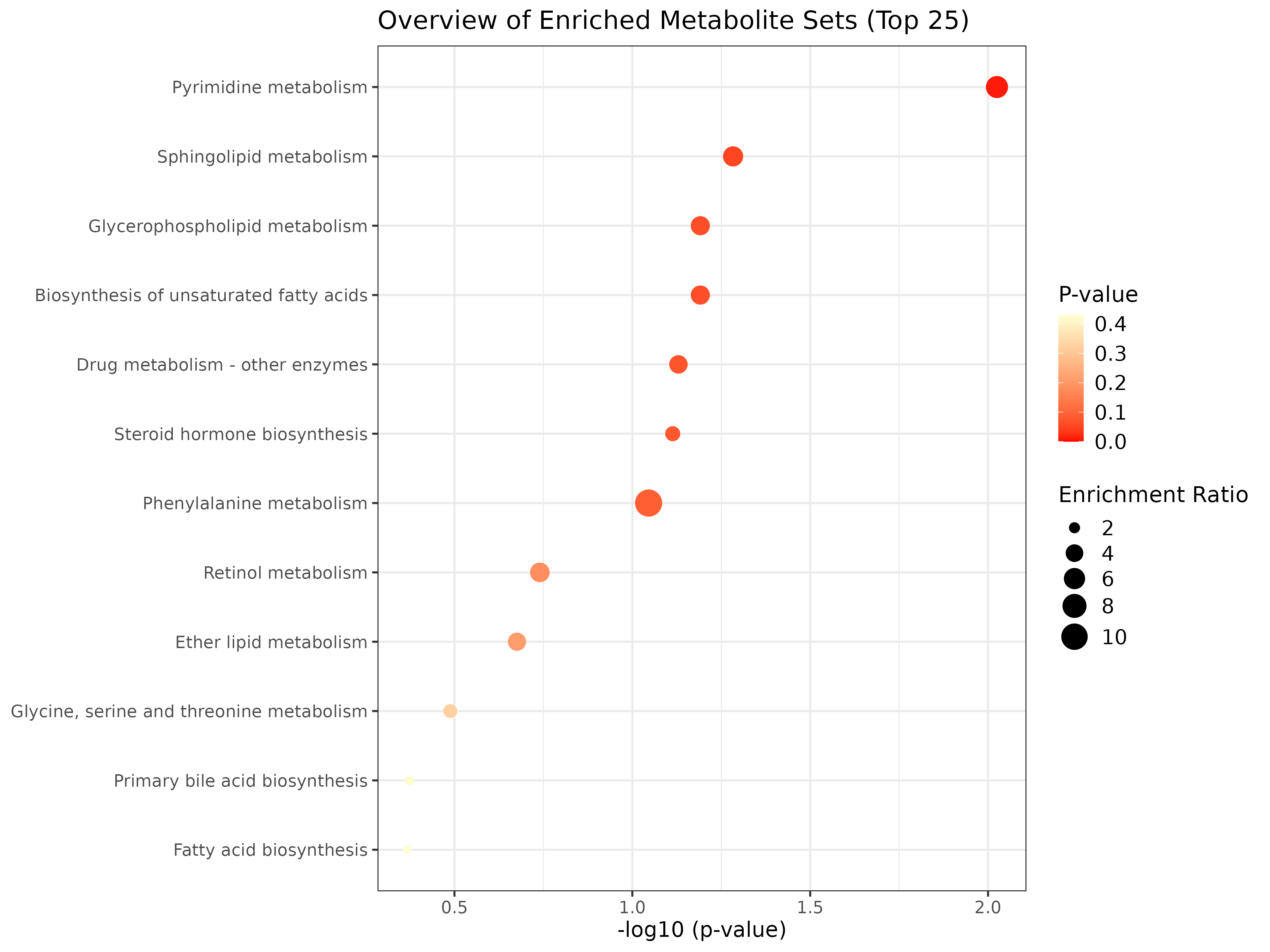

Supplement: Supplementary file 4 [file Image2.jpeg]
