# Supplementary material for: Integrated serum metabolomics and network pharmacology reveal molecular mechanism of Qixue Huazheng formula on peritoneal fibrosis
Source: Front Pharmacol. 2025 Jan 23;16:1515038. doi: 10.3389/fphar.2025.1515038 (PMC11799242; doi:10.3389/fphar.2025.1515038)

**Table S1.** Primer sequences for qPCR used in this research.


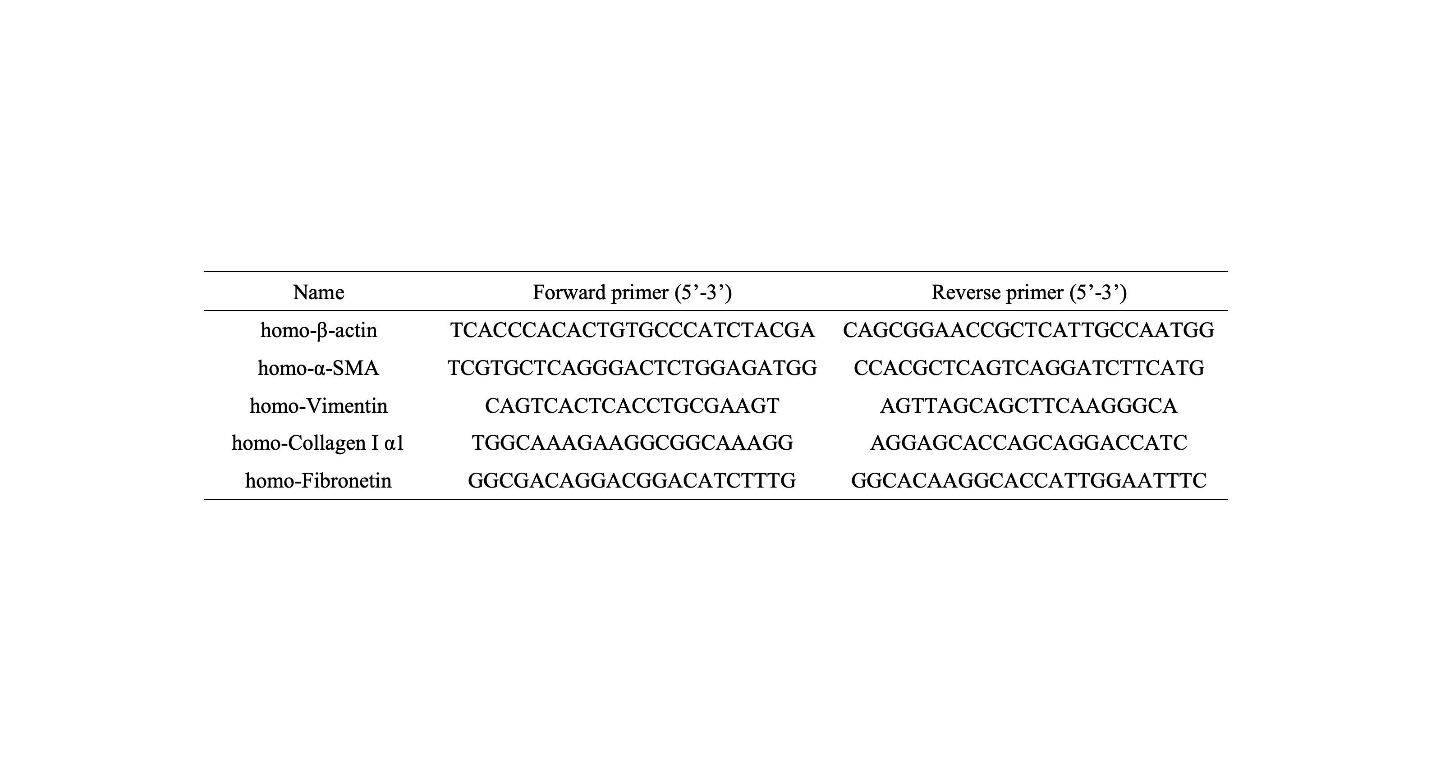


**Table S2.** The conditions of mobile phase A and mobile phase B in metabolite extraction.


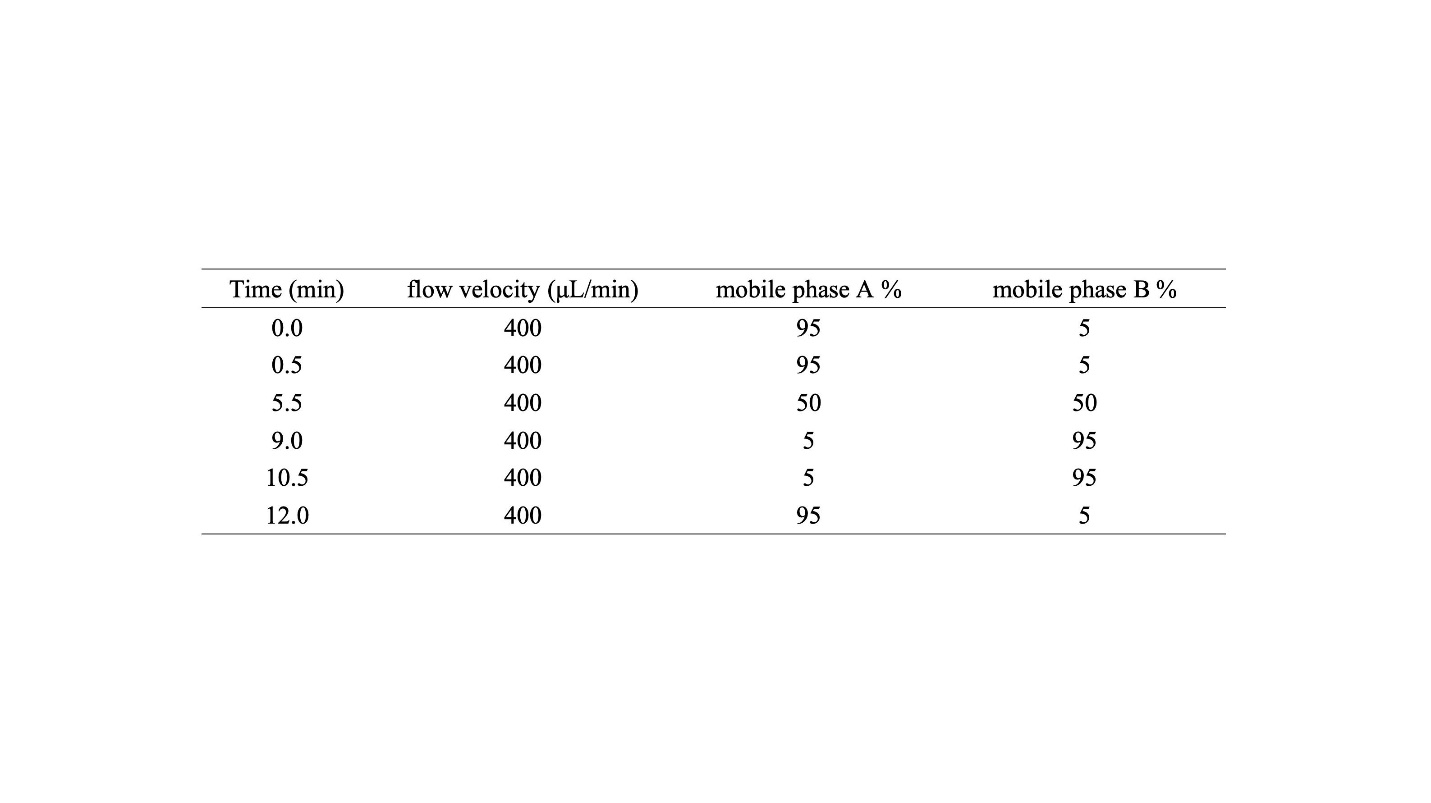


**Table S3.** The top 5 ingredients sorted by degree in compounds-targets network.


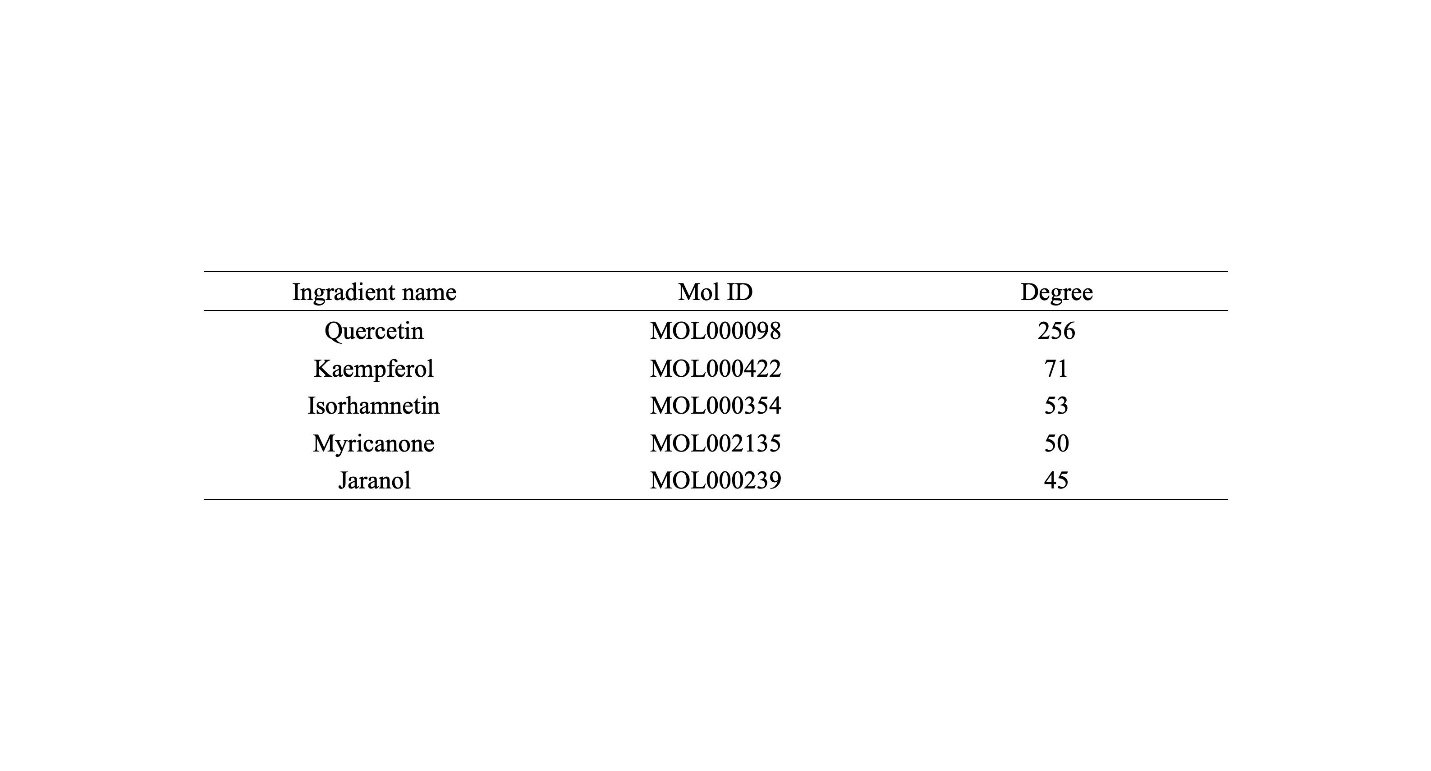


**Table S4.** The binding energies of top 5 ingredients to ESR1 and RAF1.


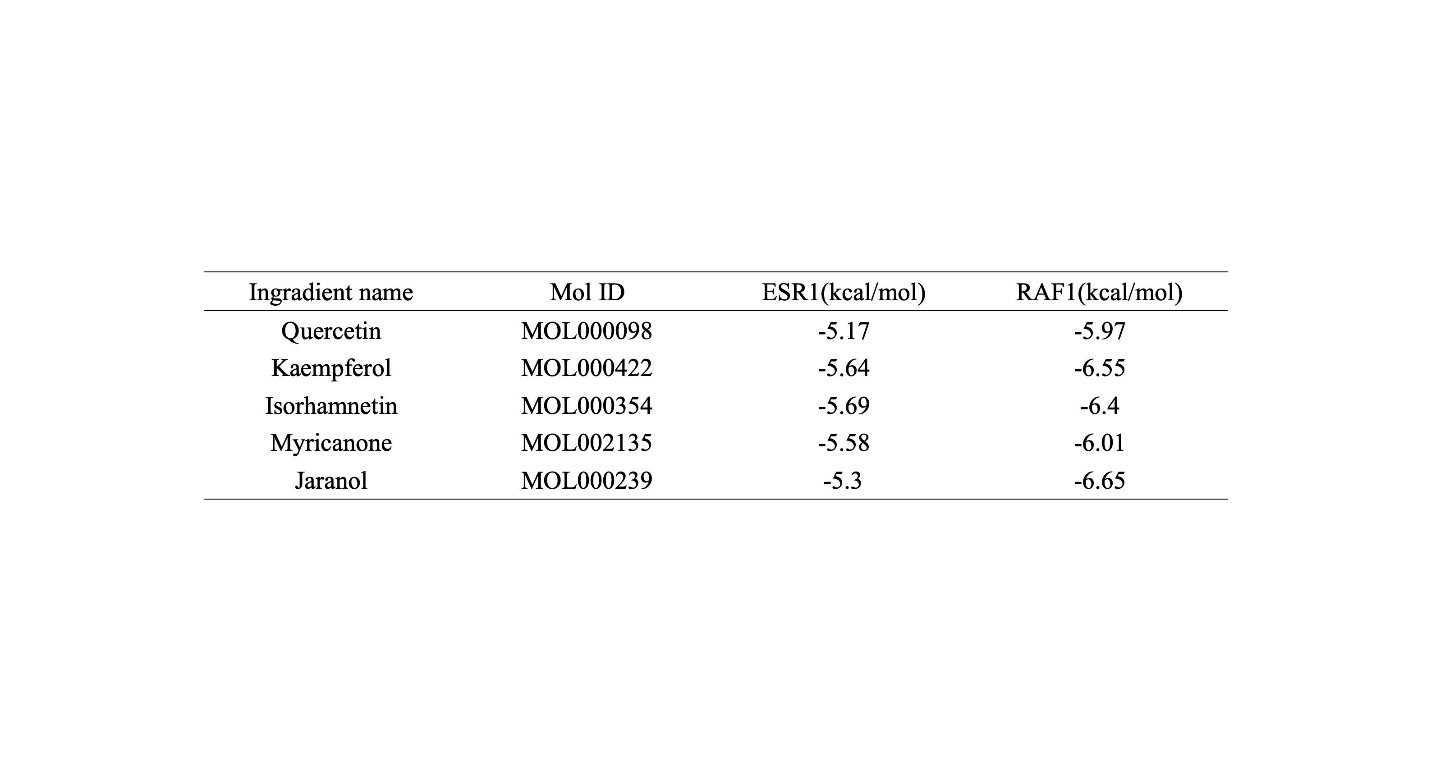

Supplement: Supplementary file 2 [file Table1.docx]
